# When the Organization Feels Like Ours: Monetary and Non-Monetary Incentives, Collective Psychological Ownership and Job Satisfaction in the Social Work Setting

**DOI:** 10.3390/bs16101734

**Published:** 2026-09-23

**Authors:** Chaoxin Jiang, Guowei Wan, Jiaqi Xi, Yu Chen, Zeyuan Cao, Xue Zhang

**Affiliations:** 1 School of Social Development, East China Normal University, Shanghai 200241, China; 2 School of Sociology and Political Science, Shanghai University, Shanghai 200444, China

**Keywords:** social worker, incentive practice, collective psychological ownership, job satisfaction, Chinese context

## Abstract

Incentive arrangements are widely employed to promote workforce stability in social work, yet limited research has systematically examined how different types of incentives are associated with job satisfaction and through what mechanisms these associations are manifested. This study aims to investigate the relationships between monetary and non-monetary incentive practices and social workers’ job satisfaction, with particular attention to the mediating roles of shared decision-making and shared hardship endurance within collective psychological ownership. The analytical sample comprises 6296 social workers from 58 cities. Mediation analyses with bootstrapped confidence intervals indicate that both monetary and non-monetary incentives are positively associated with job satisfaction, with non-monetary incentives demonstrating stronger associations. In addition, both shared decision-making and shared hardship endurance function as significant mediating pathways linking incentive practices with job satisfaction, with the shared hardship endurance dimension exhibiting a more pronounced mediating role. For social work agencies, the findings suggest combining financial rewards with professional development and team-building opportunities. Regular staff consultations and peer-support arrangements may further support shared decision-making and collective responses to organizational difficulties.

## 1. Introduction

Social work is a human service profession conducted within complex and continually changing practice environments. Social workers are frequently required to address interconnected challenges such as poverty, child abuse, domestic violence, and psychological distress (Mihai et al., 2025). These responsibilities make social work a demanding occupation in the human services sector. Their work is typically characterized by high job demands, heavy caseloads that are often constrained by bureaucratic requirements, and recurrent role conflict. Such conditions have been consistently associated with elevated levels of stress, burnout, and psychological distress among practitioners (Parlalis & Hadjicharalambous, 2024). These circumstances create an ongoing organizational challenge, particularly with respect to sustaining social workers’ commitment while ensuring the continuity and quality of services.

In response to these challenges, social work organizations commonly adopt incentive practices to support employee motivation and retention. In this study, monetary incentives encompass direct financial rewards and indirect financial benefits, whereas non-monetary incentives emphasize developmental and relational opportunities, including training, promotion, and team-building activities (Bernini & Tampieri, 2026; Dilmi et al., 2025). The relationship between work rewards and job satisfaction has a longstanding research foundation. Early studies distinguished economic returns from intrinsic work characteristics and social relationships, demonstrating the substantial importance of nonfinancial aspects of employment for job satisfaction (Kalleberg, 1977; Mottaz, 1985). Subsequent research has developed this understanding by examining meaningful work alongside the monetary and non-monetary attributes of employment (Bernini & Tampieri, 2026; Cassar & Meier, 2018). Building on this established evidence, a further question concerns how incentive practices relate to employees’ experiences of organizational membership and, through these experiences, to their evaluations of work. Collective psychological ownership (CPO) provides a framework for examining these relational meanings. CPO refers to a shared sense that an organization is collectively “ours,” encompassing perceived participation in organizational affairs and shared responsibility for its continuation (Chen & Liang, 2024; Su & Ng, 2019; Su et al., 2021). Within social work organizations, incentive provision may be associated with employees’ sense of having a voice in organizational affairs and a shared stake in the organization’s future. These experiences are reflected in two dimensions of CPO: shared decision-making and shared hardship endurance (Jiang & Jiang, 2023). Examining these dimensions together may clarify the distinct aspects of perceived shared ownership through which incentive practices are associated with job satisfaction.

Against this background, the present study examines CPO as a potential link between incentive practices and job satisfaction among Chinese social workers, with particular attention to shared decision-making and shared hardship endurance. Three research questions guide the analysis. First, are monetary and non-monetary incentive practices positively associated with social workers’ job satisfaction? Second, do shared decision-making and shared hardship endurance each account for an indirect association between incentive practices and job satisfaction? Third, which incentive category exhibits a stronger association with job satisfaction?

## 2. Literature Review

### 2.1. Incentive Practices and Job Satisfaction

Job satisfaction is commonly defined as employees’ overall affective and cognitive evaluation of their work, including satisfaction with compensation, job content, working hours, promotion prospects, and relationships with supervisors and colleagues (Bernini & Tampieri, 2026; Gazioglu & Tansel, 2006). In social work, it serves as a central indicator of how practitioners experience and appraise their professional roles. From the perspective of social exchange theory, the relationship between social workers and their employing organizations rests on reciprocal exchanges in which organizations provide resources that sustain the quality of this relationship (Cropanzano & Mitchell, 2005). Incentive practices constitute an important channel through which agencies deliver both economic and socioemotional resources to their staff (Blau, 1964). When agencies provide timely and equitable remuneration, create opportunities for training and career advancement, and recognize professional contributions, social workers are more likely to regard the employment relationship as trustworthy and fair. In accordance with the norm of reciprocity, such perceptions are typically accompanied by more positive job attitudes, of which job satisfaction is one of the most direct and central outcomes (Wayne et al., 1997). 

Although existing research has demonstrated associations between incentive practices and job satisfaction, several gaps in the literature warrant attention. First, relatively few studies have drawn a systematic distinction between monetary and non-monetary incentives. Some research has examined specific elements such as pay or perceived organizational support, but incentives are more often treated as a single, undifferentiated construct. Consequently, the distinct and combined roles of monetary incentives, such as wages and performance bonuses, and non-monetary incentives, such as promotion opportunities, training, and career development, have rarely been examined in an integrated manner (Hussein et al., 2022; Silarova et al., 2022). Second, prior studies conducted in particular national contexts have often relied on small samples drawn from limited geographic areas or single organizations, which constrains the representativeness and broader applicability of their findings (Tu et al., 2022; Xie et al., 2021). Third, the mechanisms through which incentive practices relate to job satisfaction remain insufficiently explored. In particular, the potential mediating role of collective psychological ownership has received limited attention. 

### 2.2. The Mediating Role of Collective Psychological Ownership

In recent years, CPO has received increasing scholarly attention (Chen & Liang, 2024; Jiang & Jiang, 2023; Su et al., 2021). In collectivist cultural contexts, particularly those shaped by Chinese traditions, collective interests, cohesion, and shared values are emphasized, and cooperative effort is regarded as essential to the well-being of the organization and its members (Arat & Kerelian, 2019; Jaw et al., 2007). Such orientations may shape how employees understand their relationships with the organization and with colleagues. Empirical studies have identified CPO as an important psychological resource associated with social workers’ burnout, job satisfaction, work engagement, and turnover intention (Chen & Liang, 2024; Su et al., 2021; Wei & Zhang, 2025). These findings underscore the theoretical and practical relevance of CPO in social work, a field marked by high stress, intensive interpersonal interaction, and strong reliance on teamwork.

Self-determination theory (SDT), a theory of human motivation and well-being, identifies relatedness as a universal and fundamental psychological need that involves a sense of belonging and emotional connectedness with others (Pahlevan Sharif et al., 2023). Within social service organizations, relatedness often finds expression in shared identification with the organization and in a collective perception that members jointly belong to and contribute to it, a view that is consistent with the notion of collective psychological ownership. Building on this perspective, scholars have developed CPO measures tailored to social service organizations and have conceptualized CPO as comprising two core dimensions: shared decision-making and shared hardship endurance (Su & Ng, 2019). Shared decision-making refers to the extent to which employees participate in organizational decision processes, whereas shared hardship endurance captures their willingness to persist and to assume shared responsibility when the organization encounters difficulties (Ng & Su, 2018).

Drawing on job demands-resources (JD-R) theory, incentive practices can be understood as important job resources that are linked to employees’ identification with organizational goals and values, to a stronger sense of belonging, and to the shared perception that the organization is collectively owned (Liang et al., 2021; Su et al., 2022a). Opportunities that support skill development and career advancement have been associated with stronger affective organizational commitment among social workers (Borrego & Orgambídez, 2024). Empirical findings further indicate that perceived access to training, performance-based rewards, and career development opportunities corresponds with greater affective commitment and emotional attachment to the organization (Suhail et al., 2025). In addition, team-building and other collective activities have been associated with stronger team cohesion and a more pronounced sense of shared experience among organizational members (Boštjančič et al., 2018).

Beyond its antecedents, CPO has been consistently linked to social workers’ job satisfaction. Research on CPO and related constructs indicates that employees’ sense of belonging and psychological empowerment are associated with more positive evaluations of their jobs (Pathak & Srivastava, 2020). In collectivist cultural contexts, CPO is closely intertwined with understandings of the organization as a community and with shared commitment to collective well-being (Martinović & Verkuyten, 2024). Higher levels of CPO are also associated with more stable work attitudes during periods of organizational strain, lower turnover intention, and higher job satisfaction (Su et al., 2021). When employees’ needs for emotional connection and belonging are more fully met, job satisfaction tends to be higher (Du et al., 2025). Similarly, greater opportunities for participation in decision-making and expression of professional perspectives in the workplace are associated with higher job satisfaction (Aslan & Atesoglu, 2021). Providing frontline social workers with opportunities to engage in decision-making at both project and organizational levels is therefore closely associated with more positive job evaluations (Geisler et al., 2019).

### 2.3. The Present Study

The present study integrates Social Exchange Theory, Job Demands–Resources Theory, and Self-Determination Theory to examine the associations between incentive practices and social workers’ job satisfaction. Social Exchange Theory provides the overarching relational framework, linking organizational investment in employees with favorable job attitudes (Cropanzano & Mitchell, 2005). Within this framework, JD-R Theory conceptualizes incentives as material, developmental, and social resources that support the attainment of work goals and help practitioners cope with occupational demands (Su et al., 2022a). SDT complements these perspectives by explaining how professional development, participation in decisions, and collaborative relationships may support competence, autonomy, and relatedness, respectively (Ryan & Deci, 2000). Taken together, these perspectives suggest that incentives matter through both resource provision and employees’ interpretations of organizational membership. CPO captures these relational meanings through the sense that the organization is collectively “ours.” Incentives may communicate recognition and inclusion, thereby supporting perceived participation in organizational affairs and shared responsibility for the organization’s continuation. Shared decision-making and shared hardship endurance therefore represent two proposed pathways linking incentives with job satisfaction (Su & Ng, 2019). Both incentive types are expected to show positive total associations with job satisfaction, with a stronger association anticipated for non-monetary incentives given the importance of professional development and collaborative relationships in social work (Cassar & Meier, 2018; Jessen, 2010). Figure 1 illustrates the conceptual model and the hypothesized relationships among the study variables. Accordingly, the following hypotheses are proposed:

**Hypothesis 1.** 
*Monetary incentives and non-monetary incentives are positively related to social workers’ job satisfaction.*

**Hypothesis 2.** 
*Non-monetary incentives are more strongly positively related to social workers’ job satisfaction than monetary incentives.*

**Hypothesis 3a.** 
*Monetary incentives are positively associated with job satisfaction through the shared decision-making dimension of collective psychological ownership.*

**Hypothesis 3b.** 
*Monetary incentives are positively associated with job satisfaction through the shared hardship endurance dimension of collective psychological ownership.*

**Hypothesis 4a.** 
*Non-monetary incentives are positively associated with job satisfaction through the shared decision-making dimension of collective psychological ownership.*

**Hypothesis 4b.** 
*Non-monetary incentives are positively associated with job satisfaction through the shared hardship endurance dimension of collective psychological ownership.*

## 3. Method

### 3.1. Data and Sample

This study drew on data from the 2022 wave of the China Social Work Longitudinal Study (CSWLS), a nationwide longitudinal survey examining the professional development and working conditions of social workers in China. City selection for the baseline sampling frame considered the number of social work agencies, administrative status, and socioeconomic development, encompassing major cities, provincial capitals, and additional cities in the Yangtze River and Pearl River deltas (Yuan et al., 2021). The 2022 wave yielded 6499 valid questionnaires from frontline and managerial social workers. Following the exclusion of observations with missing data on key variables, 6296 respondents were retained in the final analytical sample. Geographically, the analytical sample spanned 58 cities across mainland China, with Guangzhou (7.21%), Shenzhen (5.29%), and Nanjing (5.27%) contributing the largest city-level shares. Women constituted 81.13% of the sample, and participants had a mean age of 32.19 years (*SD* = 7.46). Frontline practitioners accounted for 62.52% of respondents, while the remaining 37.48% held supervisory or managerial positions. Detailed sociodemographic characteristics of the analytical sample are presented in Table 1.

### 3.2. Measurement

#### 3.2.1. Incentive Practices

Incentive practices were assessed by asking social workers to indicate which types of incentive mechanisms had been implemented in their employing agencies. The measure captured two distinct but complementary dimensions: Monetary Incentives and Non-monetary Incentives. The Monetary Incentives dimension comprised three items reflecting material rewards, including performance bonuses, paid leave, and educational subsidies. The Non-monetary Incentives dimension also consisted of three items, representing developmental and relational rewards such as training opportunities, promotion opportunities, and team-building activities. Each item was dichotomously coded as 0 (not implemented) or 1 (implemented). For both dimensions, total scores ranged from 0 to 3, with higher values indicating that the agency had adopted a broader array of incentive practices.

#### 3.2.2. Collective Psychological Ownership

Collective psychological ownership was measured using the Collective Psychological Ownership Scale developed by Su and Ng (2019), which has demonstrated excellent reliability and construct validity in previous empirical research (Jiang & Jiang, 2023; Su et al., 2022b). The scale consists of two theoretically distinct but related dimensions, namely shared decision-making and shared hardship endurance, which together capture employees’ collective sense of ownership within their organizations (Liang et al., 2021). Participants indicated their level of agreement with each statement on a four-point Likert scale ranging from 1 (“hardly feel this way”) to 4 (“strongly feel this way”). The shared decision-making dimension includes three items, such as “My colleagues feel that they are involved in decision-making processes,” reflecting perceptions of participatory involvement in organizational governance. The shared hardship endurance dimension also contains three items, such as “I would choose to remain even if my organization were facing difficult times,” representing employees’ commitment and perseverance during adversity. In the present study, both dimensions demonstrated satisfactory internal consistency, with Cronbach’s α = 0.817 for shared decision-making and Cronbach’s α = 0.830 for shared hardship endurance, indicating strong measurement reliability.

#### 3.2.3. Job Satisfaction

Job satisfaction was assessed using the Chinese version of the Job Satisfaction Scale developed for social workers (N. Zhang & He, 2022). This instrument captures multiple dimensions of satisfaction with one’s professional role and work environment, encompassing perceptions of job stability, compensation, opportunities for skill utilization, and interpersonal relationships at work. Participants indicated the extent of their agreement with each statement on a five-point Likert scale ranging from 1 (“strongly disagree”) to 5 (“strongly agree”). A composite score was computed by averaging responses across the ten items, with higher scores reflecting greater overall job satisfaction. In the present study, the scale demonstrated excellent internal consistency, with Cronbach’s α = 0.869, supporting its reliability for use among social workers in the Chinese context.

#### 3.2.4. Covariates

To account for potential confounding, we adjusted for eight sociodemographic and occupational characteristics selected with reference to previous research (Chen & Liang, 2024; Jiang & Jiang, 2023). Of these, five were coded as binary variables: gender (0 = male, 1 = female), ethnicity (0 = ethnic minority, 1 = Han), household registration (0 = rural, 1 = urban), religious belief (0 = no, 1 = yes), and job position (0 = non-frontline, 1 = frontline). Non-frontline positions comprised supervisory or managerial roles. Educational level was treated as an ordinal variable with four categories: 1 = junior high school or below, 2 = senior high school, 3 = junior college, and 4 = bachelor’s degree or above. Age, measured in years, and average daily working hours were included as continuous covariates.

### 3.3. Analytical Plan

The data analysis was performed using Stata 18.0, and the analytical procedures were carried out in three sequential stages. First, descriptive statistics and Pearson’s correlation analyses were conducted to examine the means, standard deviations, and bivariate associations among all study variables, including incentive practices, collective psychological ownership, and job satisfaction. This preliminary step provided an overview of variable distributions and initial evidence of interrelationships among the key constructs. Second, mediation regression analyses were employed to test the hypothesized indirect pathways from incentive practices to job satisfaction through collective psychological ownership. All models statistically controlled for gender, age, educational level, job position, ethnicity, religious belief, household registration, and daily working hours to minimize potential confounding influences. Third, the bootstrapping method with 5000 resamples was applied to estimate the confidence intervals of the indirect effects. Mediation effects were considered statistically significant when the 95% bias-corrected confidence interval did not include zero (Preacher & Hayes, 2008). This nonparametric resampling technique provides a more accurate and robust estimation of indirect effects compared with conventional methods that assume normality.

## 4. Results

### 4.1. Preliminary Analyses

Table 2 presents the descriptive statistics and bivariate correlations among the study variables. As shown in Table 2, all study variables were significantly correlated in the expected directions. Both monetary incentives and non-monetary incentives were positively associated with shared decision-making (*r* = 0.107, *p* < 0.001; *r* = 0.110, *p* < 0.001), shared hardship endurance (r = 0.167, *p* < 0.001; *r* = 0.203, *p* < 0.001), and job satisfaction (*r* = 0.166, *p* < 0.001; *r* = 0.201, *p* < 0.001). Moreover, shared decision-making and shared hardship endurance were positively correlated with job satisfaction (*r* = 0.289, *p* < 0.001; *r* = 0.520, *p* < 0.001).

### 4.2. Hypothesis Testing

Table 3 presents the results of the mediation regression models testing the indirect effects of incentive practices on job satisfaction through collective psychological ownership. In Model 1, both monetary incentives (*b* = 0.029, *p* = 0.009) and non-monetary incentives (*b* = 0.033, *p* = 0.002) were positively associated with shared decision-making, suggesting that social workers who received more incentive practices were more likely to perceive participation in organizational decision-making processes. In Model 2, both monetary incentives (*b* = 0.067, *p* < 0.001) and non-monetary incentives (*b* = 0.109, *p* < 0.001) were significantly related to shared hardship endurance, indicating that incentive practices enhance social workers’ collective commitment to persist through organizational difficulties. In Model 3, after both mediators were incorporated, monetary incentives (*b* = 0.021, *p* = 0.002) and non-monetary incentives (*b* = 0.037, *p* < 0.001) continued to exert significant positive effects on job satisfaction, supporting a pattern of partial mediation. Furthermore, both shared decision-making (*b* = 0.063, *p* < 0.001) and shared hardship endurance (*b* = 0.309, *p* < 0.001) were significant predictors of job satisfaction, confirming their mediating roles in the association between incentive practices and job satisfaction. Overall, the final model accounted for 29.8% of the variance in job satisfaction, indicating strong explanatory power and empirical support for the hypothesized mediation framework.

To further verify the mediating effects, a bootstrapping procedure with 5000 resamples was employed to estimate the indirect effects and their corresponding 95% bias-corrected confidence intervals. As presented in Table 4, both monetary incentives (β = 0.079, 95% CI [0.052, 0.106]) and non-monetary incentives (β = 0.143, 95% CI [0.115, 0.171]) exerted significant total effects on job satisfaction, with non-monetary incentives exhibiting a larger estimated total effect. Analysis of the indirect pathways revealed that both shared decision-making and shared hardship endurance significantly mediated the associations between incentive practices and job satisfaction. The indirect effects transmitted through shared decision-making were small yet statistically significant for both monetary (β = 0.003, 95% CI [0.001, 0.006]) and non-monetary incentives (β = 0.004, 95% CI [0.001, 0.007]). In contrast, the indirect effects through shared hardship endurance were substantially larger and remained statistically significant for both monetary (β = 0.037, 95% CI [0.024, 0.050]) and non-monetary incentives (β = 0.066, 95% CI [0.053, 0.079]). Overall, the bootstrapping results provide strong empirical support for the dual-mediation model of collective psychological ownership, reinforcing the robustness of the proposed hypotheses. 

## 5. Discussion

Drawing on data from 6296 social workers in 58 cities across mainland China, this study examined the associations between incentive practices and social workers’ job satisfaction, with particular attention to the mediating roles of the two dimensions of collective psychological ownership. The findings indicate that incentive practices are positively associated with social workers’ job satisfaction overall. In addition, non-monetary incentives demonstrate a stronger association with job satisfaction than monetary incentives. The results further suggest that both shared decision-making and shared hardship endurance are significant mediating mechanisms through which incentive practices are related to job satisfaction. 

Consistent with prior research and the expectations guiding this study, the findings indicate that incentive practices are positively associated with Chinese social workers’ job satisfaction. From a social exchange perspective, employees may interpret incentive practices as signals that their efforts are reciprocated and their contributions are recognized, which may in turn correspond with more positive evaluations of their organization and work (Blau, 1964; Cropanzano & Mitchell, 2005). Employees who perceive that they are treated fairly and valued by the organization tend to report higher job satisfaction (Cropanzano & Mitchell, 2005; Rhoades & Eisenberger, 2002). In Chinese social work, where practitioners often operate under heavy workloads and constrained resources, experiences of organizational support and recognition may be particularly salient in shaping overall job evaluations (Xie et al., 2021). Existing research on Chinese social workers has largely concentrated on adverse occupational outcomes, such as burnout, stress, and turnover intention, whereas comparatively few studies have examined how incentive practices relate to job satisfaction. By foregrounding job satisfaction as a key occupational outcome, this study offers a more comprehensive perspective on social workers’ work experiences. In addition, the large, multi-city sample extends prior research, which has often relied on smaller or single-organization samples, and provides more robust empirical evidence on the associations between incentive practices and job satisfaction.

The finding that non-monetary incentives exhibit stronger associations with job satisfaction than monetary incentives underscores the importance of non-monetary job attributes in helping professions. This pattern is consistent with earlier research highlighting the importance of intrinsic and social rewards for work satisfaction within social service settings (Borzaga & Tortia, 2006; Jessen, 2010). At the level of individual psychological experience, non-monetary incentives may be more readily interpreted as expressions of organizational recognition and respect for employees’ professional contributions (Ryan & Deci, 2000; Y. Zhang et al., 2024). Such recognition may strengthen positive perceptions of the work environment and correspond to higher overall job satisfaction. At the level of professional values and work meaning, social work is strongly shaped by commitments to service, social contribution, and professional identity. Under these conditions, job attributes related to meaningfulness, professional development, and participation in organizational life may carry particular weight in employees’ overall evaluations of their work. Prior research has likewise shown that meaningful work and opportunities for professional growth are closely associated with higher job satisfaction and that non-pecuniary aspects of work play an important role in shaping employees’ job evaluations. The present findings are consistent with this line of research and suggest that non-monetary forms of organizational support may be especially salient in relational and mission-oriented occupations (Cassar & Meier, 2018).

A further contribution of this study lies in extending research on CPO as a psychosocial resource by examining how its two dimensions link monetary and non-monetary incentive practices with job satisfaction (Chen & Liang, 2024; Su et al., 2022b). Shared decision-making captures perceived participation in organizational affairs, whereas shared hardship endurance reflects shared responsibility for sustaining the organization through difficulties (Su & Ng, 2019). Examining both dimensions within the same model provides a more differentiated account of the relational meanings attached to organizational rewards. Specifically, the findings suggest that incentive provision is connected to job satisfaction through employees’ experiences of participation and their commitment to a shared organizational endeavor. The larger estimated indirect effects through shared hardship endurance for both incentive types warrant further theoretical interpretation. From a social exchange perspective, organizational investment may communicate recognition and support, encouraging employees to reciprocate through commitment to the organization and willingness to share its difficulties (Cropanzano & Mitchell, 2005). In emotionally demanding service settings, such shared commitment may carry particular significance because practitioners depend on continuing cooperation to address workplace challenges. JD-R Theory helps explain why collective orientations of this kind may operate as psychological resources within demanding work environments (Su et al., 2022a). SDT offers a complementary interpretation: perceived participation in decisions may support autonomy, whereas commitment to facing difficulties together may carry meanings of solidarity and belonging relevant to relatedness (Ryan & Deci, 2000). The latter may be especially salient in practitioners’ evaluations of work when emotional demands and resource constraints make experiences of collective support particularly valuable. A similar distinction between the two dimensions has been reported in the association between role stress and job performance among Chinese child welfare workers (Jiang & Jiang, 2023). The present study extends this distinction to organizational incentive provision and job satisfaction, suggesting that participation and shared responsibility may differ in their relevance to how employees evaluate organizational rewards, and thereby provides a more specific relational account of workforce motivation in social work. 

### 5.1. Theoretical and Practical Implications

Theoretically, this study advances a relational understanding of job satisfaction by examining collective psychological ownership as a pathway connecting incentive practices with social workers’ evaluations of their work. Distinguishing shared decision-making from shared hardship endurance highlights two complementary aspects of perceived shared ownership: participation in organizational affairs and shared responsibility for navigating organizational difficulties. The larger estimated indirect effects through shared hardship endurance suggest that mutual commitment under challenging circumstances may be particularly relevant in emotionally demanding service settings. Alongside these relational processes, the stronger association observed for non-monetary incentives reinforces the importance of developmental and social attributes of employment. Taken together, these findings extend understanding of how organizational rewards and experiences of shared ownership relate to job satisfaction in professions characterized by collaborative practice and strong professional values.

From a practical perspective, the findings suggest that social service agencies could integrate incentive provision with participatory and supportive management. Agency human resource policies could combine adequate financial compensation with structured training, transparent promotion opportunities, and team-building activities, giving sustained attention to both economic and non-economic aspects of employment (Bernini & Tampieri, 2026; Cassar & Meier, 2018; Gazioglu & Tansel, 2006). Such arrangements could support shared decision-making by involving practitioners in decisions about service delivery, workload allocation, and professional development. Regular case discussions, peer consultation, and supportive supervision may further enable staff to exchange expertise and address difficulties collectively (Barth et al., 2008; Osborne et al., 2020). Team-level recognition and performance feedback may also acknowledge shared contributions and reinforce the collective meaning of organizational rewards (Pierce et al., 2026). To support shared hardship endurance, expectations of collective responsibility should be accompanied by tangible managerial support, adequate resources, and manageable workloads. Embedding these practices in everyday management may strengthen employees’ experiences of participation and mutual support within the organization (Borrego & Orgambídez, 2024; Jiang & Jiang, 2023).

At the policy level, these organizational practices can be supported through stable funding arrangements and clearer workforce provisions in social service commissioning. Given that many social work services in China are project-based and purchased by the government, policymakers could incorporate explicit provisions for staff remuneration, professional development, and supportive supervision into service contracts, together with appropriate budgetary support (Wen, 2017). Minimum standards for monetary and non-monetary support could provide a common basis for incentive provision across agencies, while predictable funding could facilitate sustained implementation. Regular assessments could monitor employees’ access to incentives, opportunities to participate in organizational decisions, and perceptions of workplace support, alongside job satisfaction (Huebner & Zacher, 2021). This information could inform adjustments to funding criteria and management requirements, positioning workforce development and employee participation as integral components of social service policy.

### 5.2. Limitations and Future Research

Several limitations of this study should be acknowledged. First, the cross-sectional nature of the data precludes firm conclusions regarding causal relationships among incentive practices, collective psychological ownership, and job satisfaction. Although the findings are consistent with the proposed framework, longitudinal or experimental designs would be valuable for clarifying temporal sequencing and strengthening causal interpretation. Second, all variables were derived from social workers’ self-reports, which may be subject to common method variance and social desirability bias. Subsequent studies could strengthen the robustness of the evidence by incorporating multiple data sources, such as supervisor ratings, administrative records, or peer assessments, to provide a more comprehensive picture of workplace processes. Third, the study is situated within the Chinese social work context, where institutional arrangements and cultural norms may shape the relevance and expression of collective psychological ownership. Further research is therefore needed to examine the cultural applicability and generalizability of collective psychological ownership and its associations with incentive practices and job satisfaction in social work samples from other national and cultural settings.

## 6. Conclusions

This study examined how monetary and non-monetary incentives relate to job satisfaction among Chinese social workers, considering the mediating roles of two dimensions of collective psychological ownership. Both incentive types were positively associated with job satisfaction, with non-monetary incentives showing a larger estimated total association. Significant indirect associations were observed through shared decision-making and shared hardship endurance, with larger estimates for the latter. These findings extend research on work rewards by highlighting perceived participation and shared responsibility as relational processes relevant to job satisfaction. For social service management and policymaking, they suggest integrating financial and developmental support with meaningful staff participation and organizational arrangements that enable practitioners to address workplace difficulties collectively.

## Figures and Tables

**Figure 1:**
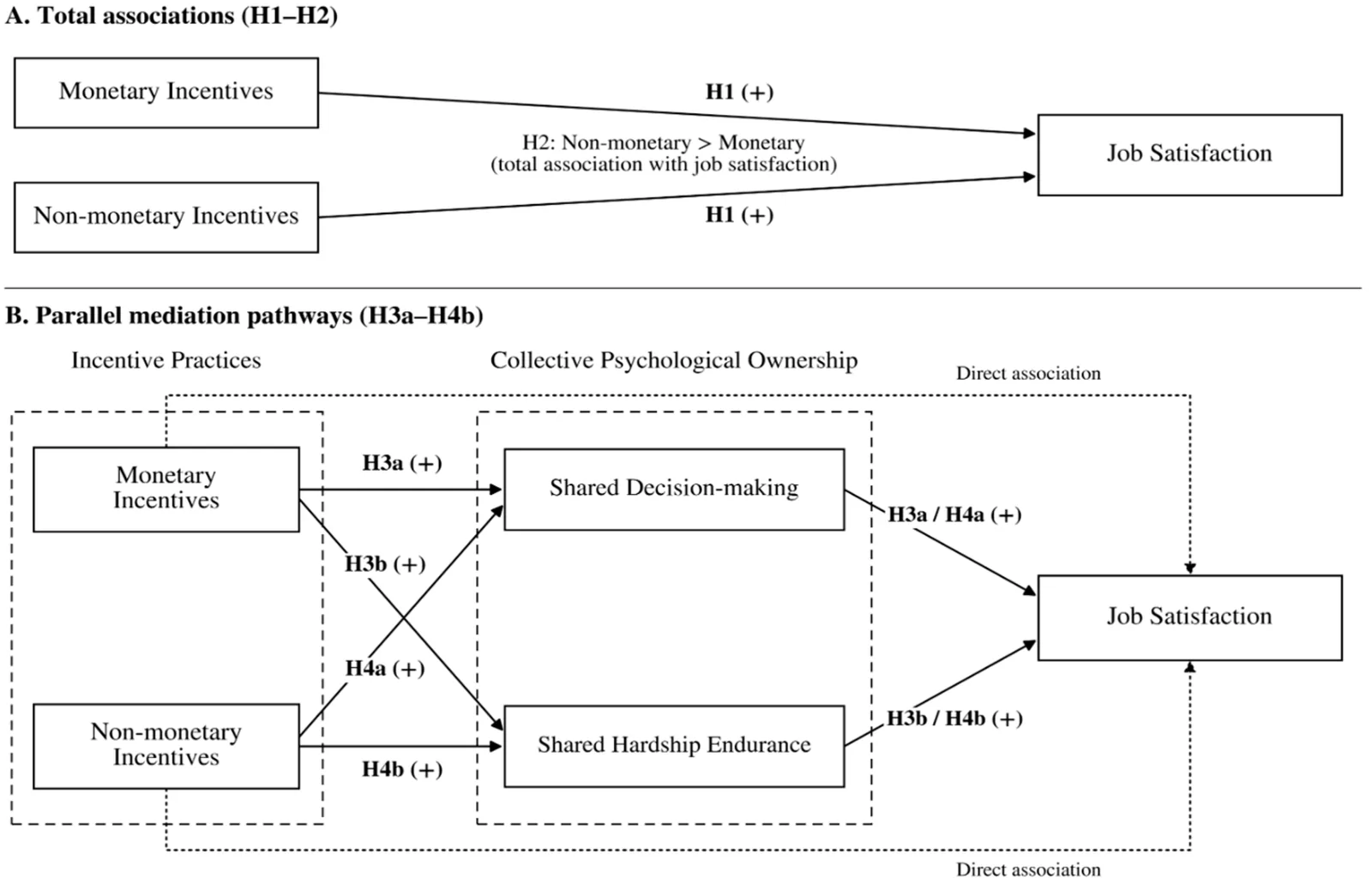
Conceptual framework.

**Table 1:** Sociodemographic characteristics of respondents (*N* = 6296).

Sociodemographic Characteristics	Percentage/Mean (*SD*)
Gender	
Male	18.87
Female	81.13
Age	32.19 (7.46)
Educational level	
Junior high school or below	0.43
Senior high school	5.40
Junior college	28.24
Bachelor’s degree or above	65.93
Job position	
Social workers with management or supervisory roles	37.48
Frontline workers without management or supervisory roles	62.52
Ethnicity	
Han ethnicity	92.90
Ethnic minority	7.10
Household registration	
Urban	59.20
Rural	40.80
Religious belief	
Have	3.53
Not have	96.47
Daily working hours	7.84 (1.25)

**Table 2:** Descriptive statistics and correlations of study variables.

	Mean	*SD*	1	2	3	4	5
1. Monetary incentives	1.110	0.952	1.000				
2. Non-monetary incentives	1.826	1.043	0.518 ***	1.000			
3. Shared decision-making	1.837	0.758	0.107 ***	0.110 ***	1.000		
4. Shared hardship endurance	2.502	0.765	0.167 ***	0.203 ***	0.397 ***	1.000	
5. Job satisfaction	3.588	0.530	0.166 ***	0.201 ***	0.289 ***	0.520 ***	1.000

Note: *** *p* < 0.001.

**Table 3:** Results of the mediation model.

Variable	Model 1: Shared Decision-Making			Model 2: Shared Hardship Endurance			Model 3: Job Satisfaction		
	*b*	*SE*	*p*	*b*	*SE*	*p*	*b*	*SE*	*p*
Gender	−0.208	0.024	<0.001	−0.032	0.024	0.183	−0.021	0.015	0.139
Age	0.007	0.001	<0.001	0.013	0.001	<0.001	0.006	0.001	<0.001
Educational level	0.050	0.016	<0.001	−0.094	0.016	<0.001	−0.016	0.010	0.105
Job position	−0.258	0.020	<0.001	−0.087	0.020	<0.001	−0.030	0.012	0.014
Ethnicity	−0.166	0.036	<0.001	−0.085	0.036	0.020	0.027	0.022	0.213
Religious belief	0.067	0.050	0.185	0.022	0.051	0.662	−0.039	0.031	0.207
Household registration	0.001	0.020	0.955	0.020	0.020	0.323	−0.001	0.012	0.964
Daily working hours	0.021	0.007	0.005	−0.005	0.007	0.543	−0.012	0.005	0.010
Monetary incentives	0.029	0.011	0.009	0.067	0.011	<0.001	0.021	0.007	0.002
Non-monetary incentives	0.033	0.010	0.002	0.109	0.010	<0.001	0.037	0.006	<0.001
Shared decision-making							0.063	0.008	<0.001
Shared hardship endurance							0.309	0.008	<0.001
*R* ^2^	0.071			0.077			0.298		
*F*	48.02 ***			52.76 ***			222.56 ***		

Note: *** *p* < 0.001.

**Table 4:** Results of bootstrapping analysis.

	β	Boot *SE*	95% Confidence Interval	
			Lower	Upper
Total effect				
Monetary incentives → Job Satisfaction	0.079	0.014	0.052	0.106
Non-monetary incentives → Job Satisfaction	0.143	0.014	0.115	0.171
Direct effect				
Monetary incentives → Job Satisfaction	0.038	0.012	0.015	0.062
Non-monetary incentives → Job Satisfaction	0.073	0.013	0.047	0.098
Indirect effect				
Monetary incentives → Shared decision-making → Job Satisfaction	0.003	0.001	0.001	0.006
Non-monetary incentives → Shared decision-making → Job Satisfaction	0.004	0.001	0.001	0.007
Monetary incentives → Shared hardship endurance → Job Satisfaction	0.037	0.006	0.024	0.050
Non-monetary incentives → Shared hardship endurance → Job Satisfaction	0.066	0.007	0.053	0.079

## Data Availability

The datasets presented in this article are not readily available because the data are from the 2022 China Social Work Longitudinal Study (CSWLS) and the authors are not authorized to publicly share or redistribute them. Requests to access the datasets should be directed to the CSWLS research team.
